# A resource for automated search and collation of geochemical datasets from journal supplements

**DOI:** 10.1038/s41597-022-01730-7

**Published:** 2022-11-25

**Authors:** Erin L. Martin, Vitor R. Barrote, Peter A. Cawood

**Affiliations:** School of Earth, Atmosphere and Environment, Monash University, Clayton, Victoria 3800 Australia

**Keywords:** Geochemistry, Databases

## Abstract

This article presents a resource for automated search, extraction and collation of geochemical and geochronological data from the Figshare repository using web scraping code. To answer fundamental questions about the Earth’s evolution, such as spatial and temporal evolution and interrelationships between the planet’s solid and surficial reservoirs, researchers must utilize global geochemical datasets. Due to the volume of data being published, these datasets become quickly outdated. We present a resource that allows researchers to rapidly curate and update their own databases from existing published data. We use open-source Python code to web scrape the Figshare repository for journal supplementary files using the application programming interface, allowing for the collection and download of hundreds of supplementary files and metadata in minutes. Use of this web scraping tool is demonstrated here by collation of a zircon geochronology and chemistry database of >150,000 analyses. The database is consistent in reproducing trends in other published zircon compilations. Providing a resource for automated collection of Figshare data files will encourage data sharing and reuse.

## Abstract (summary)

**Table:** 

Measurement(s)	age and chemistry of zircon
Technology Type(s)	mass spectrometry

## Background & Summary

**Fig. 1:**
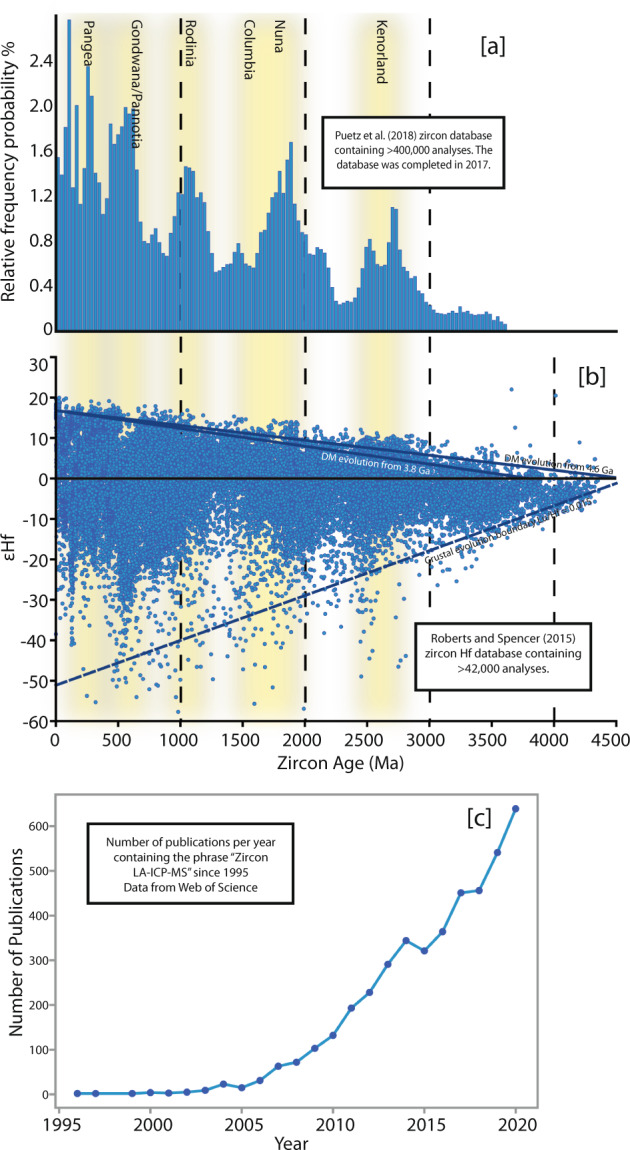
(**a**) Zircon database compiled by Puetz, *et al*.^4^ in 2017, containing >400,000 analyses. (**b**) Zircon Hf database compiled by Roberts and Spencer^5^. (**c**) Web of Science data on publications containing the phrase “Zircon LA-ICP-MS” between 1995 and 2020. From 2015-2020 (i.e. since the Roberts and Spencer database was published), more than 2300 zircon LA-ICP-MS articles have been published.

Geoscience research increasingly relies on geochemical, geochronologic, or isotopic data to investigate Earth processes in deep time. Increasingly, articles are utilizing big-data to establish statistically sound secular trends and unravel large-scale, multi-system changes in the Earth^1–11^. This has coincided with instrumentation developments (primarily in the field of Laser Ablation Inductively Coupled Mass Spectrometry (LA-ICP-MS)) which allow for rapid production of large datasets (several hundred unknown analyses per day), translating to a rapid rise in the rate of data generation and publication (Fig. 1).

The uptick in data production and use of published datasets in geology research has been accompanied by the publication of global zircon geochronology and geochemical databases. Many zircon (or other mineral phase) geochronologic, isotopic or geochemical databases occur as single published entities, which, given the rate of data production are quickly outdated. For example, since the Puetz^12^ zircon U-Pb database was compiled in 2017, over 1600 articles containing “zircon LA-ICP-MS” have been published (Fig. 1). While there are a growing number of permanent repositories available (for example, AusGeochem, EarthChem, GEOROC, StratDB), the supplementary data to journal articles remains an important source of geochronological and geochemical data used in creating databases and new publications. The rapid increase in data production has not been matched by publication of studies to automate the data collection process, despite the push for an increase in robustness of statistical data treatment and exploration into the use of machine learning to solve geoscience problems^4,13,14^.

Academic journal publishers are implementing supplementary data file hosting on large repositories. For example, Mendeley Data hosts supplementary material for Elsevier Journals, while Figshare^15–17^ hosts data from Earth Science publications in journals from Springer Nature, The Geological Society of London, The Geological Society of America, and Taylor & Francis. Such repositories are important data resources and allow researchers to use automated techniques to search and collect data such as web scraping.

Web scraping is the method of extracting and saving data from the internet to a file or database for later use, and can be conducted either manually or by using a program or bot^18^. Web scraping is commonly used for collecting data from social media platforms, price comparison of items and stocks, monitoring website changes, monitoring weather data and to build internet search engine responses^19^. Some web scraping programs crawl the internet parsing the HyperText Markup Language (HTML) of the website. These methods are often discouraged or banned by specific websites due to the interruptions to normal web services that can occur through data retrieval of this kind. Further, repeated service requests by HTML web crawlers or scrapers mimic the function of some malicious hacking software^18^, and many websites will block the source IP address of such attempts, rendering web scraping code useless.

Rather than encouraging HTML web scraping, many websites, including data repository sites such as Figshare, provide Application Programming Interfaces (APIs) to enable interaction of programs or bots with a set of services at the back-end of the website, to avoid affecting the front-end functionality. APIs act as a service that provides a high-level interface to directly retrieve data from their repositories or databases at the back-end^18,19^.

Here, we present open-source code to allow for fast automated collection of geochemical and geochronological data from supplementary files of published journal articles using API web scraping of the Figshare repository. Using this method, we generated a dataset of ~150,000 zircon U-Pb, Lu-Hf, REE, and oxygen isotope analyses. This code gives researchers the ability to quickly compile the relevant supplementary material to build their own geochemical database and regularly update it, whether their data of interest be common or niche. Automation of the data collection process through web scraping may remove bias in data collection that is introduced through manual author searches and allows researchers to efficiently search and collect relevant geochemical and geochronological data for database curation.

## Methods

Using the Figshare API web scraping code written in Python programming language, we compiled a dataset of zircon analyses. The zircon dataset presented here is comprised of U-Pb, Lu-Hf, REE and oxygen isotope data from multiple scrapes of the Figshare database using search terms *zircon Lu-Hf*, *zircon hafnium*, *zircon* *rare earth elements*, *zircon trace elements*, *zircon REE, zircon oxygen* and *zircon oxygen isotopes*. In total, ~600 articles were returned in search responses and ~1000 supplementary files associated with these articles were downloaded. Not all of these supplementary files contained relevant zircon data (some included detailed methodology, additional figures, etc), but zircon data from >500 supplementary files were ultimately incorporated into the database.

**Fig. 2:**
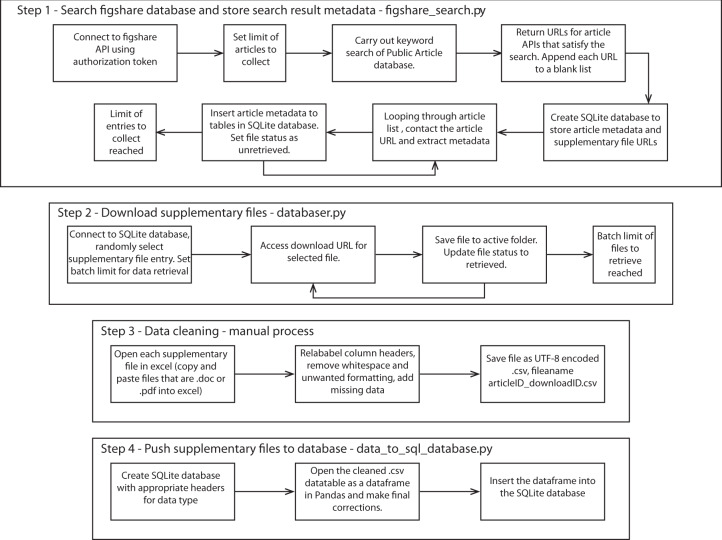
Workflow of the steps involved in creating the zircon database through web scraping the Figshare public article database.

**Fig. 3:**
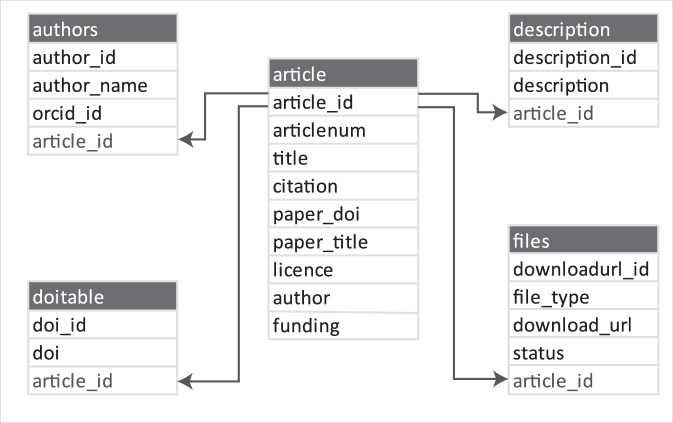
Relational database schema for the collection of scraped metadata, created in Step 1 of the workflow shown in Fig. 2.

The database was created in four steps outlined by the workflow in Fig. 2. First, the program establishes a connection with the Figshare API and carries out a search of the Figshare Public article database based on a keyword, or set of keywords input by the user. The user sets the number of results to retrieve, and the article API URLs that satisfy the search criteria are appended to a list. The program then creates or opens an existing SQL database, and iterating through the list of article URLs, extracts and stores the metadata for each article. The metadata collected includes the unique Figshare article number, title, citation, data DOI, publication DOI, publication title, license, article description, and download URL for each of the supplementary files associated with the article (Fig. 3). All data in the GEOSCRAPE database falls under one of three Creative Commons licenses (CC0, CC-BY-4.0, or CC BY-NC) the most restrictive of which allows for attributed, non-commercial re-use of the data. The compiled dataset is published by the most restrictive license within the dataset.

The second part of the Python code (Fig. 2, Step 2) iterates through the SQL database and downloads unretrieved files to the working folder. The user sets the number of files to be retrieved by a single execution of the code (Fig. 2, Step 3). Following downloading the files and before they can be entered into a geochemical database, the data require cleaning. This process includes reformatting and relabelling data, as well as including missing data and metadata so that the data can be interpreted correctly. The data cleaning process is the most time-consuming step of the workflow. This is a practical example of issues caused by bad data reporting and poor data citation practices in geochemistry. Minimum metadata requirements for geochemical data have been defined^20^ and have started to be adopted and disseminated^21^, however, have yet to become the prevalent standard of practice. Furthermore, standardized formatting would facilitate the cleaning process, as discussed in the Technical Validation section.

Once the data have been cleaned, they can be pushed into an SQL database designed by the user. We have used the open-source software SQLite to create the database in Python for zircon geochronology and geochemistry. Calculated values (with the exception of U-Pb or Pb-Pb ages), for example, εHf model ages and concordia ages have not been included in the database to limit the metadata required (i.e. decay constants) to interpret the data.

As SQLite is not generally used as a data editing or interrogation tool, using it in combination with Python coding allows for secure storage of data as SQLite. Once the database has been compiled, it can easily be exported to a .csv file from the SQLite graphical user interface, or through Python. The .csv file can then be interrogated, modified and updated in Python or Excel. A modified version of the database .csv (for example with additional columns including calculated data) and be imported to SQLite to create a new database or data table within the same database. Storage of the database in SQLite and editing of an exported .csv means that a master copy of the data is retained in the event that the .csv is corrupted or damaged in any way. Additionally, the SQLite platform allows for relational databases to be built to store data if the database is extremely large or complex. For the sake of simplicity and the likelihood that many researchers are unfamiliar with relational databases and SQL coding, we have created a database that is a single table in SQLite. This is available on Figshare (see Code Availablilty section for details).

## Data Records

The GEOSCRAPE^22^ database contains zircon geochronological, isotopic, and chemical data from >260 published articles. There are >150,000 entries in the database, the majority of those are radiometric age data. The database includes the unique Figshare article ID and DOI of the publication the data was contained within, minimizing the possibility of duplicate data in the large database. The inclusion of other published data compilations within this database was avoided to further reduce data duplication risks.

**Fig. 4:**
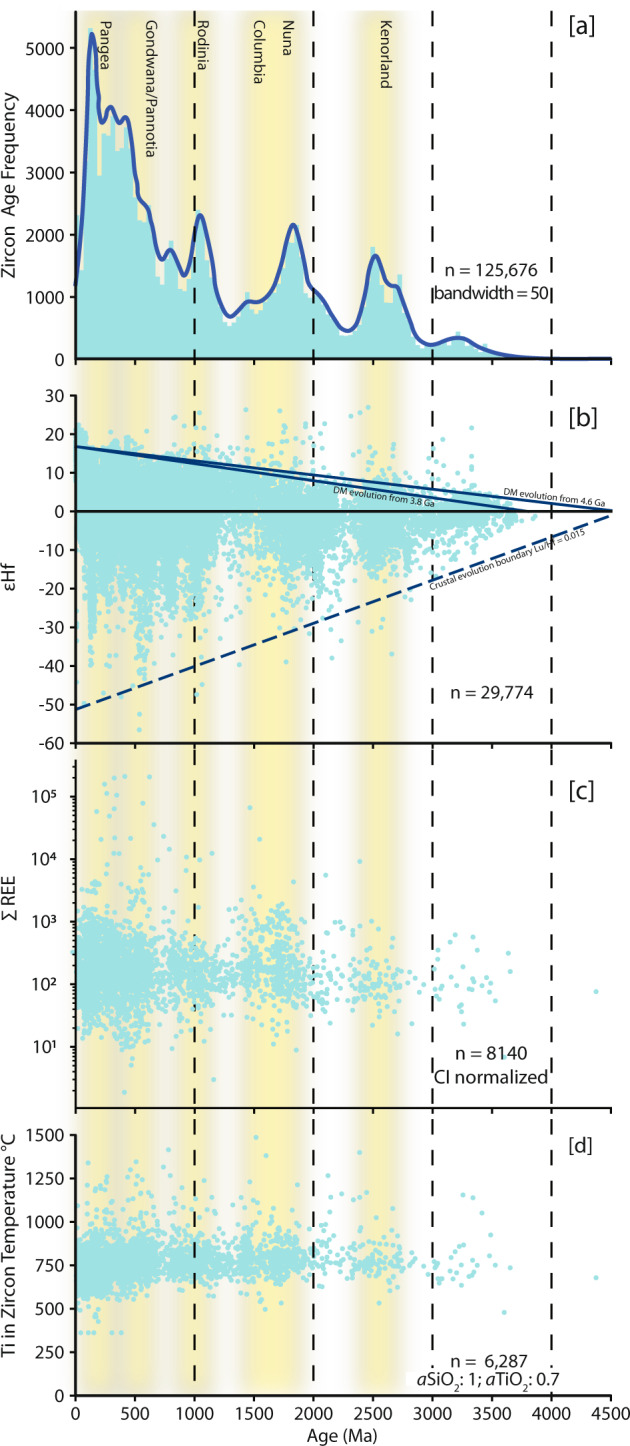
Zircon data compilation figures created from the GEOSCRAPE^22^ database. (**a**) Frequency histogram and kernel density estimate of zircon ages (Ma). (**b**) ɛHf versus zircon age scatter plot. DM: Depleted mantle. Constants used in calculation: Lu decay after Scherer, *et al*.^232^; Chondrite Uniform reservoir ratios after Bouvier, *et al*.^233^; Depleted mantle ratios Griffin, *et al*.^234^. (**c**) ∑ rare earth element (REE) concentrations versus zircon age. Concentrations are CI chondrite normalized using the values from Anders and Grevesse^24^ (**d**) Scatter plot of Ti-in-zircon temperatures versus zircon age, method after Ferry and Watson^25^. All plots contain both concordant and discordant age data.

Figure 4 shows a summary of zircon data compiled within the GEOSCRAPE^22^ database. Frequency histogram and kernel density estimate (Fig. 4a) show major zircon crystallization peaks broadly coinciding with phases of supercontinent formation (yellow bands in Fig. 4). The peaks are consistent with those produced by the Puetz^12^ database (Fig. 1a) and other zircon age compilations such as Voice, *et al*.^7^ and Cawood, *et al*.^23^ despite those datasets being compiled through manual search processes, and our compilation containing slightly fewer than half the number of zircons in the Puetz^12^ database.

Similarly, the zircon ɛHf versus zircon age plot (Fig. 4b) displays a similar distribution of data to that of Roberts and Spencer^5^, including negative ɛHf excursions at ca. 550 and 200 Ma. In contrast to the Roberts and Spencer^5^ database, which includes summary age and Hf data only, the GEOSCRAPE database includes all available geochemical, isotopic and sample information for each analysis.

Zircon trace element data were also collated in the database and the ∑ rare earth element (REE) and Ti-in-zircon temperature versus zircon age plots (Fig. 4c,d) represent a summary of those data. The >8000 REE concentrations presented in Fig. 4d are CI chondrite normalized^24^. The >6000 Ti-in-zircon temperatures plotted were calculated assuming silica saturation and an *a*TiO_2_ of 0.7 (method after Ferry and Watson^25^). All available trace and rare earth element data that resulted from the web scraping search were included in the database (currently >100,000 individual elemental analyses and >8,000 zircon rare earth element suites).

The database is openly available on the public GEOROC repository at 10.25625/FWQ7DT as an Expert Dataset and will be regularly updated by ELM until 2027. Researchers can freely access the data and use the web scraping code and database to create subsets or build further on the database without duplicating data. All analyses within the GEOSCRAPE^22^ database are linked to a DataCite DOI.

## Technical Validation

Development of the web scraping code presented here has highlighted inconsistencies in data formatting and reporting. With the increasing use of programming packages such as Matlab, R and Python for data processing and statistical analysis in Earth science, it is necessary that reporting of such data move to a format that can be easily integrated into such programs. For example, reading an isotopic data table into such a program should result in allocation of an isotopic ratio as a column header, i.e. ^207^Pb/^206^Pb. However, many authors use shorthand notation to label their data including (observed during compilation of this database): 207/206, 7/6, Pb207/Pb206, Pb/Pb, or format column headers across multiple rows. This creates difficulties when attempting to use automation to correctly identify data within a table, as tools like regular expressions require some predictability in the formatting of strings for searching.

Most published databases are formatted in a fashion that requires manual data cleaning before the data can be read as a table into a Matlab, R or Python. Formatting issues encountered include: data published in .jpeg^26^, .pdf or .doc formats, or within the body of the article^27^, rather than .xls, .tab or .csv; blank space at the top of the table; column headings separated across multiple rows; merged cells, and; variation between vertical and horizontal formatting (e.g., a single row including sample ID, sample description, sampling coordinates, followed by the analyses row by row). These issues substantially increase the time and effort it takes for published data to be re-used and increase the likelihood of introducing errors into the data. Publication of data tables in .pdf or .jpeg formats, or solely within the body of an article discourages the re-use of those data, possibly leading to bias in large datasets due to data exclusion and should be avoided.

Metadata that is essential for the re-use of published geochemical datasets are often not included within the supplementary data tables and requires additional investigation of the associated article to retrieve it. Examples of such data are sampling coordinates, which are substantially under-reported, reported with incomplete information such as UTM data with no zone reported^28^, regional grid references with no reference to a global coordinate system provided^29^, or simply inferred through sampling locations plotted on maps^30,31^ which require georeferencing to accurately extract the coordinates. Other often absent metadata include analytical methods and parameters utilized, analyzed phases, any standard quality assurance/quality control procedures, and the type of uncertainty reported.

Inadequate data attribution is also becoming prevalent during publication of data compilations when citations for the original data are restricted to the supplementary data or appendices. Removal of data citations from the references section of a manuscript may be inadvertently encouraged by journals with strict limits on the number of references that may be cited.

**Fig. 5:**
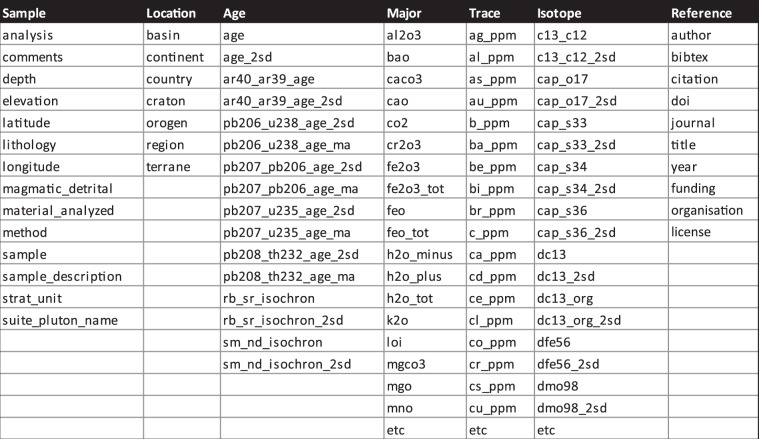
Example of column headers for supplementary data tables. Table S1 shows the extended table of preferred column headers. etc: etcetera.

Here we propose a set of guidelines for the formatting of supplementary data tables that will allow for published data to be more readily utilized by the community:

- Reporting of data following guidelines similar to those of Horstwood, *et al*.^32^
- Sample identification via International Geo Sample Number and sample metadata after Chamberlain, *et al*.^21^ and Broman and Woo^33^
- Include all metadata required to understand, interpret and use the data tables as a single entity
- Ensure each column header is unique; i.e. do not use ‘±’ for columns with uncertainty, report the type and level of uncertainty, e.g. 2sd, 1sd, 1SE, 1σ
- Contribute data in either comma-delimited or tab-delimited formats
- Column headers must be restricted to one row in the data table
- Use underscores rather than spaces in column headers
- Limit the use of special characters
- Use UTF-8 encoding when saving .csv files to ensure errors are not introduced when reading the file into other programs
- Include sampling coordinates for each sample in a globally recognized coordinate system such as WGS1984, or UTM with all zone details
- Where possible, include each variable in a separate column and each analysis in a separate row. Avoid blank spaces
- Do not use merged cell formatting
- Report the method for common Pb correction calculation, when applicable (as well as uncorrected data)
- Report normalization factors and constants used in a separate column or at the bottom of the data table
- Figure 5 lists possible column headers that may be required for a variety of geochemical datasets following Gard, *et al*.^10^ and Lehnert, *et al*.^34^

Duplication of data in large data compilations is a source of concern. To minimize this in the dataset, any data syntheses in data tables (e.g. any analysis that gave a citation to a previous publication) were not included in the dataset. Further, during data cleaning, repeated data tables were discluded from the dataset. These were reasonably simple to identify as they often had consecutive article IDs, or the metadata for the article could be looked up in the data sources table produced during Step 1 of the web scraping code. It is likely that a small amount of human error has resulted in some data duplication in the dataset. However, we estimate it to be less than 1%.

## Data Availability

The web scraping and data-basing code used in this paper are available from https://github.com/erinlmartin/figshare_geoscrape.git. Instructions for use are available with the code. All data presented in this study are previously published and available on the Figshare repository. DOIs for the data are included within the GEOSCRAPE^22^ database hosted with GEOROC at: 10.25625/FWQ7DT. This database will be updated by ELM until 2027. Table S1, SQLite template files^35^ and metadata table available from Figshare: 10.6084/m9.figshare.16870603.v3.
